# Spatial optimization for radiation therapy of brain tumours

**DOI:** 10.1371/journal.pone.0217354

**Published:** 2019-06-28

**Authors:** Cameron Meaney, Marek Stastna, Mehran Kardar, Mohammad Kohandel

**Affiliations:** 1 Department of Applied Mathematics, University of Waterloo, Waterloo, Ontario, Canada N2L 3G1; 2 Department of Physics, Massachusetts Institute of Technology, Cambridge, Massachusetts, United States of America 02139; University of California Irvine, UNITED STATES

## Abstract

Glioblastomas are the most common primary brain tumours. They are known for their highly aggressive growth and invasion, leading to short survival times. Treatments for glioblastomas commonly involve a combination of surgical intervention, chemotherapy, and external beam radiation therapy (XRT). Previous works have not only successfully modelled the natural growth of glioblastomas *in vivo*, but also show potential for the prediction of response to radiation prior to treatment. This suggests that the efficacy of XRT can be optimized before treatment in order to yield longer survival times. However, while current efforts focus on optimal scheduling of radiotherapy treatment, they do not include a similarly sophisticated spatial optimization. In an effort to improve XRT, we present a method for the spatial optimization of radiation profiles. We expand upon previous results in the general problem and examine the more physically reasonable cases of 1-step and 2-step radiation profiles during the first and second XRT fractions. The results show that by including spatial optimization in XRT, while retaining a constant prescribed total dose amount, we are able to increase the total cell kill from the clinically-applied uniform case.

## Introduction

Glioblastomas are the most aggressive, and unfortunately most common, form of primary brain tumour [1–5]. They are characterized by rapid growth and invasiveness, yielding survival times that seldom exceed a year [6]. Because of this, treatments for glioblastomas are swift and aggressive, usually involving a combination of surgical intervention, chemotherapy, and external beam radiation therapy (XRT). Furthermore, the tendency for recurrence of glioblastomas after surgery makes postoperative chemotherapy and XRT a crucial part of effective treatments. Although current treatment plans do often extend survival time, they are far from perfect and leave much room for improvement. However, while these efforts focus on optimal scheduling of radiotherapy, they do not include a similarly sophisticated spatial optimization.

Non-uniform dose distributions are not a new concept in radiation oncology. Phenotypic variations across the volume of a tumour can result in differing levels of radiation effectiveness. In particular, hypoxic regions cause a difference in cell radio-sensitivity, making radiation less effective in those areas. This leads to a technique called “dose-painting” in which different regions are prescribed a different dose to combat the reduced effect (see [7, 8] for an overview). Dose painting divides the tumour into a small, discrete number of regions allowing a different dose to be prescribed to each area. Additionally, we may need to apply non-uniform radiation to areas with different cell density, irrespective of radio-sensitivity.

Several previous works have addressed the dependence of the optimal beam shape on the density profile of the tumour, (see [9–12] for example). A possible criterion for optimality (used in our calculation) is minimizing the total number (*N*) of tumour cells remaining after application of XRT; another is to minimize the tumour control probability (TCP), *e*^−*N*^ (see below, Eq (7)). Naturally either minimization must satisfy a number of physical constraints. The constraint on the total radiation dose (used in the paper) leads to a particular shape of the beam, which was also obtained in previous studies [9, 11, 12]. By contrast, a constraint on the ‘mean dose,’ weighted by the local cell density, leads to a uniform beam profile [10].

In this paper, we study the following question: given a maximum allowable total dose to administer, what dose profile results in the maximum global TCP. As an equivalent metric to the TCP, we instead minimize the total number of surviving tumour cells after radiotherapy. We then go further, making our conclusions clinically reasonable by discretizing this optimal profile and considering multiple radiation fractions. While we do not deal with tumour heterogeneity in particular, we do include an alternative death mechanism which can account for different radio-sensitivies resulting from tumour heterogeneity such as hypoxia. Other works have examined optimization of radiation therapy incorporating normal tissue complication probability (NTCP) in addition to TCP [8, 13]. The NTCP is an important measure to consider as it quantifies the chance of problems arising in nearby organs at risk. While we do not include NTCP in this work, an interesting topic for future study would be to incorporate TCP and NTCP in a single mathematical optimization.

Naturally, when discussing optimization in a given system, mathematical modelling is an invaluable tool. In the context of brain tumours, mathematical models have been widely used by many different investigators (for example, [14–18]). While originally developed for investigations of brain tumours, we anticipate that these models and the following approach and results can be easily generalized to study other types of tumours. In the following, we build upon a host of previous works (see [19] for a review) which show that the natural growth of glioblastomas can be well-described by two governing parameters, *ρ* and *D*_*n*_, which describe growth and invasion processes respectively. The two mathematical models most commonly used to describe growth of tumours are the so-called exponential and logistic growth laws. The more general logistic growth naturally provides a better description of tumour proliferation and stabilization. However, during the relatively short time frame before the growth of the tumour begins to plateau, exponential growth is a reasonable approximation. Including a linear (Laplacian) diffusion term, commonly used to model tumour invasion, we arrive at the well-established equation for tumour growth

$$\begin{array}{c} \frac{\partial n(\vec{x},t)}{\partial t}=D_{n}\nabla^{2}n\left(\vec{x},t\right)+\rho n\left(\vec{x},t\right)(1-\frac{an(\vec{x},t)}{n_{max}}). \end{array}$$ (1)

Here, $n(\vec{x},t)$ is the tumour cell density at position $\vec{x}=\left(x_{1},\cdots ,x_{d}\right)$, and $\nabla^{2}=\sum_{\alpha =1}^{d}\partial^{2}/\partial x_{\alpha }^{2}$ is the Laplacian operator. We have introduced *d* as the number of dimensions; *d* = 3 in three dimensions, while for certain computations we focus on the two dimensional case of *d* = 2. Exponential growth corresponds to *a* = 0, while *a* = 1 leads to logistic growth to a maximal density *n*_*max*_.

Prior to treatment, two MRIs are commonly performed: one diagnostic and one to aid in treatment planning. Using the measures for velocity of growth and tumour abnormality obtained from these two scans alone, the tumour-specific values for *ρ* and *D*_*n*_ can be estimated [18].

We begin by presenting the general mathematical model, followed by studies of a number of special cases. First, the optimal continuous profile is derived for the cases of one and two radiative fractions with both exponential and logistic cytotoxic action. The insights from these cases are subsequently extended to show that the optimal tumour cell density is uniform for any radiation procedure. From there, we move to optimization of non-continuous radiation profiles, and derive the optimal scenario for the cases of one-step and two-step profiles for single or multiple fractions, constrained individually or together. Finally, the non-continuous optimization is extended to include a logistic cytotoxic action for one and two step radiation profiles.

## Materials and methods

### Model derivation

Consider a tumour that has evolved according to Eq (1) for some time, such that its cell density profile is given by $n(\vec{x},t_{0})$. Now we intend to apply XRT to this tumour. To do so, we introduce a function $f(\vec{x},t)$ which we call the cytotoxic profile. The action of most therapeutic interventions is to remove a fraction of existing cells, which is incorporated in our model by adding a term $-\gamma f\left(\vec{x},t\right)n\left(\vec{x},t\right)\left(1-bn\left(\vec{x},t\right)/n_{max}\right)$ to the right hand side of Eq (1). For radiation, the parameter *γ* is a measure of the radiation rate, and can be written as $\gamma =\alpha \dot{D}$ (1/day) where *α* is the linear coefficient in the well-known Linear-Quadratic model for radiation efficacy, and $\dot{D}$ is the dose rate applied to the tumour. For simplicity, we do not include the quadratic term of the Linear-Quadratic model into the model as it does not affect the qualitative shape of the radiation profile, which is the focus of this study. This is clear mathematically since the inclusion of the quadratic component simply perturbs the value of *γ* in Eq (2) below, which acts as a scaling constant. The parameter *b*, much like *a* in the growth term, simply differentiates between exponential (*b* = 0) and logistic (*b* = 1) cell killing. It is also known that saturated tumours with a high cell-density and low proliferation are less affected by radiation than low cell-density tumours. A logistic death term is mathematically able to capture this behaviour as a higher cell density reduces the magnitude of the final term. This addition modifies the governing equation to

$$\begin{array}{c} \frac{\partial n}{\partial t}=D_{n}\nabla^{2}n+\rho n(1-\frac{an}{n_{max}})-\gamma f\left(\vec{x},t\right)n(1-\frac{bn}{n_{max}}). \end{array}$$ (2)

Normal XRT occurs in a series of bursts called fractions. We therefore impose that $f(\vec{x},t)$ is an on-again-off-again function in time such that $f(\vec{x},t)=0$ for all times except during the scheduled fractions. This greatly simplifies the solution to the nonlinear partial differential equation, as during the time interval Δ*t* over which each fraction is applied, the processes of cell division and spreading have little effect on the cell density. The natural growth of tumours proceeds on scales of days, months, or years, while radiation fractions occur over a scale of minutes. Thus, during each fraction, the first two terms on the right hand side of Eq (2) can be mathematically neglected, leading to

$$\begin{array}{c} \frac{\partial n}{\partial t}{|}_{fraction}\approx -\gamma f\left(\vec{x},t\right)n(1-\frac{bn}{n_{max}}). \end{array}$$ (3)

Integrating this (now ordinary) differential equation in the interval *t* ∈ [*t*_0_, *t*_0_ + Δ*t*], leads to

$$\begin{array}{c} n\left(\vec{x},t_{0}+\Delta t\right)=n\left(\vec{x},t_{0}\right)e^{-\gamma f(\vec{x},t_{0})\Delta t}, \end{array}$$ (4)

in the exponential case (*b* = 0), and

$$\begin{array}{c} n\left(\vec{x},t_{0}+\Delta t\right)=\frac{n_{max}}{1-(\frac{n\left(\vec{x},t_{0}\right)-n_{max}}{n(\vec{x},t_{0})})e^{-\gamma f(\vec{x},t_{0})\Delta t}}, \end{array}$$ (5)

in the logistic case (*b* = 1). We focus on the first and second fractions of XRT and further impose a simple upper bound for $f(\vec{x},t)$ to adhere to patient safety standards. We write this constraint as $0\leq f(\vec{x},t)\leq C$ for some *C*. The final constraint on $f(\vec{x},t)$ limits the total dose received by the patient. This constraint is mathematically represented by

$$\begin{array}{c} \gamma \int d^{d}\vec{x}\;dt\;f\left(\vec{x},t\right)\leq F\;, \end{array}$$ (6)

where the time integral is over the entire treatment length. This constrains the total radiation dose over all fractions (where we expect the inequalities to be saturated to achieve maximal effectiveness).

Our goal is to determine the function $f(\vec{x},t)$ that minimizes the total number of tumour cells after the final fraction, *N*(*T*), obtained by integrating the tumour cell density as

$$\begin{array}{c} N\left(T\right)=\int d^{d}\vec{x}\;n\left(\vec{x},T\right). \end{array}$$ (7)

To contrast, Brahme and Agren [9] instead optimized the TCP, which they defined as (using our notation) TCP = *e*^−*N*(*T*)^ where *N*(*T*) is the number of cells surviving the treatment. Regarding $n(\vec{x},t_{0}+\Delta t)$ as the mean of a probability density for cells following XRT, TCP is the probability that all cells are exterminated. Clearly, one can see that minimizing *N*(*T*) is equivalent to maximizing *e*^−*N*(*T*)^. Furthermore, for the simplest case, we indeed achieve the same result.

## Results

### Continuous profile optimization

We begin by analysing continuous cytotoxic profiles for one fraction of radiation. In the case of exponential death, the result matches that of previous studies, and from it we can derive a precise shape of the profile. In the case of logistic death, the resulting profile is more complex, but remains mathematically consistent with the previous case. A similar analysis of the second fraction of radiation is significantly more challenging as the effect of normal tumour progression must be taken into account. While it is possible, the results are far less interesting since the optimal profile will leave a uniform (or flat-topped) cell density (proof of this in supporting information). The second fraction is mathematically examined in the supporting information for the case of exponential death. The second fraction for the logistic death case.

#### Optimal profile with one fraction of exponential death

To proceed in the case of exponential cytotoxic action, we impose the first constraint in Eq (6) with a Lagrange multiplier λ, which requires extremizing

$$\begin{array}{c} \tilde{N_{1}}=\int d^{d}\vec{x}\;n\left(\vec{x},t_{0}\right)e^{-f(\vec{x},t_{0})}+\lambda (\int d^{d}\vec{x}\;f\left(\vec{x},t_{0}\right)-F), \end{array}$$ (8)

where we have set *γ* = Δ*t* = 1 for convenience. Solving the resulting Euler-Lagrange equation for $f(\vec{x},t_{0})$, we find the optimal profile

$$\begin{array}{c} f\left(\vec{x},t_{0}\right)=\text{ln}[\frac{n(\vec{x},t_{0})}{\lambda }], \end{array}$$ (9)

with λ chosen such that Eq (6) is satisfied. Using this cytotoxic profile would give us the optimal cell kill from the radiation fraction. Not surprisingly, following the simplifications leading to Eq (9), the above result is independent of the parameters *ρ* and *D*_*n*_. The appearance of the logarithm is merely a consequence of the killing effect of therapy being proportional to the number of existing cells, with the cytotoxic profile appearing in the exponent of Eq (4).

This result has been previously derived by many others ([9–11] for example). An interesting consequence of this optimal profile is that it leaves the resulting cell density as a uniform distribution. Stavreva *et al* [10] applied an extremization of TCP subject to a constraint on the mean dose defined as $D_{v}\propto \int n\left(\vec{x},t_{0}\right)f\left(\vec{x},t_{0}\right)d^{d}x$, i.e. weighting the dose according to the local cell density. It is clear that if this constraint is used in Eq (8) in place of the unweighted net dose, the resulting beam profile will be uniform.

While a useful starting point, the cytotoxic profile in Eq (9) is not guaranteed to satisfy the constraints of $0\leq f(\vec{x},t)\leq C$. In particular, it leads to unphysical negative values when $n(\vec{x},t_{0})<\lambda$. To better understand the limitations of this result, and how to overcome them, let us consider the simplest case of a Gaussian profile arising from radially symmetric growth of a single cell in exponential growth: assuming that the tumour begins with a single oncogenic transformation or single-cell metastasis, modelled by a delta-function cell density, exponential growth for a time *t*_0_ leads to the cell density profile

$$\begin{array}{c} n\left(r,t_{0}\right)=n_{0}e^{-\frac{r^{2}}{2\sigma^{2}}}, \end{array}$$ (10)

where *r* is the radial distance from the initial cell (tumour center). The width of the Gaussian profile is $\sigma =\sqrt{2D_{n}t_{0}}$, while $n_{0}=e^{\rho t_{0}}/{\left(2\pi \sigma^{2}\right)}^{d/2}$ is the cell density at its center. Eq (9) now leads to a parabolic cytotoxic profile. Cutting off the negative portions of the parabola leads to the (semi-circular) profile

$$\begin{array}{c} f\left(\vec{x},t_{0}\right)\equiv f_{1}\left(r\right)=\text{ln}(\frac{n_{0}}{\lambda })-\frac{r^{2}}{2\sigma^{2}}\equiv \{\begin{array}{cc} f_{m}(1-\frac{r^{2}}{r_{m}^{2}})\; & \text{if}\;r\leq r_{m}\\ 0 & \text{if}\;r\geq r_{m} \end{array}. \end{array}$$ (11)

We have introduced the parameters *f*_*m*_ = ln(*n*_0_/λ) and $r_{m}=\sigma \sqrt{2f_{m}}$ to indicate the maximum value of the cytotoxic profile, and the radius over which it is applied, respectively. The total radiation dose in this fraction is then given by

$$\begin{array}{c} F=\int d^{d}\vec{x}\;f_{1}\left(r\right)=\frac{2S_{d}}{d(d+2)}f_{m}r_{m}^{d}, \end{array}$$ (12)

where *S*_*d*_ is the *d*-dimensional solid angle, with *S*_3_ = 4*π* and *S*_2_ = 2*π*. Using $f_{m}=r_{m}^{2}/\left(2\sigma^{2}\right)$ from Eq (11), we conclude that the optimal radius of the semi-circular beam is given by

$$\begin{array}{c} r_{m}=(\frac{d(d+2)}{S_{d}}{)}^{\frac{1}{d+2}}F^{\frac{1}{d+2}}\sigma^{\frac{2}{d+2}}, \end{array}$$ (13)

while its maximal intensity equals

$$\begin{array}{c} f_{m}=\frac{1}{2}(\frac{d(d+2)}{S_{d}}{)}^{\frac{2}{d+2}}F^{\frac{2}{d+2}}\sigma^{-\frac{2(d+1)}{d+2}}. \end{array}$$ (14)

If the above value of *f*_*m*_ exceeds the maximum allowed intensity of *C*, we should instead use

$$\begin{array}{c} f_{1}^{C}\left(r\right)=\{\begin{array}{cc} C-\frac{r^{2}}{2\sigma^{2}}\; & \text{if}\;r\leq r_{m}\\ 0 & \text{if}\;r\geq r_{m} \end{array}, \end{array}$$ (15)

with *r*_*m*_(*F*) now constrained by the maximal allowed density according to Eq (12). In either case, the cell density profile after application of XRT will attain a flat-top profile, as

$$\begin{array}{c} n\left(r,t_{0}+\Delta t\right)=\{\begin{array}{cc} n_{0}e^{-f_{m}}\; & \text{if}\;r\leq r_{m}\\ n(r,t_{0}) & \text{if}\;r\geq r_{m} \end{array}. \end{array}$$ (16)

A flat post-XRT profile is also predicted for any initial tumour cell density $n(\vec{x},t_{0})$, although the volume over which the beam is applied will be different. In the best outcome, the density profile will be below the (single-cell) threshold for future growth. If not, additional fractions need to be applied.

#### Optimal profile with one fraction of logistic death

Mathematically, the switch from an exponential to a logistic cell death does not change much. We now simply use Eq (5) instead of Eq (4) in our optimization. Again imposing the constraint of Eq (6) using a Lagrange multiplier and setting *γ* = Δ*t* = 1 for convenience, we arrive at the optimal profile

$$\begin{array}{c} f\left(\vec{x},t_{0}\right)=\text{ln}[(\frac{n_{max}-n\left(\vec{x},t_{0}\right)}{n(\vec{x},t_{0})})(\frac{2\lambda }{2\lambda +n_{max}+\sqrt{n_{max}\left(n_{max}+4\lambda \right)}})]\;. \end{array}$$ (17)

Like the previous single-fraction case, this result is independent of *ρ* and *D*_*n*_. It also is not guaranteed to satisfy the bounds of $0\leq f(\vec{x},t)\leq C$. As a quick check on the validity of this result, one can let *n*_*max*_ → ∞ and see that Eq (9) is recovered. Note that the optimal profile again has a uniform or flat-toped form. This can be seen by substituting Eq (17) into Eq (5).

### Discrete profile optimization

Computational methods exist for determining how to administer a heterogeneous dose, primarily *sub-volume boosting* and *dose painting by numbers* [7, 8]. Unfortunately, these methods are limited by the mathematical optimization techniques as well as the physical reasonability of their results. Specifically, current technology is not capable of producing beams with a high degree of precision for clinical use such as those derived above in the Continuous Profile Optimization. The standard procedure is to coalesce several beams on the location of the tumour, creating an area of high radiative strength. As such, a reasonably practical non-uniform beam profile is a step function. In the following, we consider cases of both one-step and two-step radiation profiles. We emphasize that our aim is to explore the feasibility of spatial optimization and to gain qualitative insights, and thus make mathematical simplifications throughout to reflect this. The first simplification is to consider *radially symmetric profiles in two dimensions*. In the following, we assume exponential growth (*a* = 0) leading to a Gaussian profile, as in Eq (10). A summary of the parameter values used in calculations can be seen in Table 1.

**Table 1:** Model parameters.

Parameter	Symbol	Value (Unit)	Reference
Initial Total Cell Number	*N*	10^7^ (cells)	This Work
Tumour Cell Diffusivity	*D*_*n*_	0.32 (mm^2^/day)	[20]
Tumour Cell Proliferation Rate	*ρ*	0.35 (1/day)	[20]
Linear-Quadratic Parameter	*α*	0.08 (1/Gy)	Average of values from [17]
Dose Rate during Radiation	$\dot{D}$	5 (Gy/fraction)	Chosen within range from [21]
Radiation Effect Parameter	*γ*	60 (1/day)	Estimated from *α* and $\dot{D}$
Dose Limiting Parameter	C	2.5 (Gy)	This Work

Model parameters used in the various calculations and simulations throughout the paper. Those with the given reference of ‘This Work’ were selected within a biologically reasonable range from various sources as an example for calculations.

#### D1: One-step radial profile

The simplest step-function case of XRT involves a uniform beam of radius *r*_1_ and strength *f*_1_, applied for a duration Δ*t* at time *t*_0_, i.e.

$$\begin{array}{c} f\left(r,t\right)=\{\begin{array}{cc} f_{1}\; & 0\leq r\leq r_{1}\;\text{and}\;t_{0}\leq t\leq t_{0}+\Delta t,\\ 0 & \text{otherwise}. \end{array} \end{array}$$ (18)

The goal is minimize *N*(*t*_0_ + Δ*t*) = 2*π* ∫ *r* *n*(*r*, *t*_0_ + Δ*t*) *dr*, subject to a constraint on *F* which we rewrite as a constraint on $F^{\prime }\equiv \frac{F}{\pi \Delta t\gamma }=r_{1}^{2}f_{1}$. Approximating the PDE as an ODE as before, the tumour cell density distribution immediately after the fraction is obtained as

$$\begin{array}{c} n\left(r,t_{0}+\Delta t\right)=\{\begin{array}{cc} n\left(r,t_{0}\right)e^{-\gamma f_{1}\Delta t} & 0\leq r\leq r_{1},\\ n(r,t_{0}) & r_{1}<r\leq R. \end{array} \end{array}$$ (19)

Integrating this result gives the total number of cells as

$$\begin{array}{cc} N(t_{0}+\Delta t) & =\;\int \int \;n(r,t_{0}+\Delta t)dA\\ & =2\pi [e^{-\gamma f_{1}\Delta t}n_{0}\int_{0}^{r_{1}}re^{-\frac{r^{2}}{2\sigma^{2}}}dr+n_{0}\int_{r_{1}}^{R}re^{-\frac{r^{2}}{2\sigma^{2}}}dr]\\ & =2\pi n_{0}\sigma^{2}[e^{-\gamma f_{1}\Delta t}(1-e^{-\frac{r_{1}^{2}}{2\sigma^{2}}})+e^{-\frac{r_{1}^{2}}{2\sigma^{2}}}-e^{-\frac{R^{2}}{2\sigma^{2}}}]. \end{array}$$ (20)

The constraint on the total beam flux can be imposed through a Lagrange multiplier λ to create an augmented *N*,

$$\begin{array}{c} \tilde{N}=2\pi n_{0}\sigma^{2}[e^{-\gamma f_{1}\Delta t}(1-e^{-\frac{r_{1}^{2}}{2\sigma^{2}}})+e^{-\frac{r_{1}^{2}}{2\sigma^{2}}}-e^{-\frac{R^{2}}{2\sigma^{2}}}]-\lambda \left(f_{1}r_{1}^{2}-F^{\prime }\right)\;. \end{array}$$ (21)

Extremizing with respect to *r*_1_, *f*_1_, and λ leads to the following system of equations:

$$0=2\pi n_{0}r_{1}[e^{-\frac{r_{1}^{2}}{2\sigma^{2}}-\gamma f_{1}\Delta t}-e^{-\frac{r_{1}^{2}}{2\sigma^{2}}}]-2\lambda f_{1}r_{1},$$ (22)

$$0=2\pi n_{0}\sigma^{2}[-\gamma \Delta te^{-\gamma f_{1}\Delta t}+\gamma \Delta te^{-\frac{r_{1}^{2}}{2\sigma^{2}}-\gamma f_{1}\Delta t}]-\lambda r_{1}^{2},$$ (23)

$$0=f_{1}r_{1}^{2}-F^{\prime }.$$ (24)

After eliminating *f*_1_ and λ from the above equations, we arrive at the following implicit expression for *r*_1_:

$$\begin{array}{c} 0=e^{-\frac{F^{\prime }\gamma \Delta t}{r_{1}^{2}}}+(\frac{r_{1}^{4}}{2F^{\prime }\gamma \Delta t\sigma^{2}}-1)e^{-\frac{r_{1}^{2}}{2\sigma^{2}}-\frac{F^{\prime }\gamma \Delta t}{r_{1}^{2}}}-\frac{r_{1}^{4}}{2F^{\prime }\gamma \Delta t\sigma^{2}}e^{-\frac{r_{1}^{2}}{2\sigma^{2}}}. \end{array}$$ (25)

Values of *r*_1_ that satisfy Eq (25) can be used to find a corresponding *f*_1_, together specifying the optimal *N*(*r*_1_, *f*_1_). We assume that the duration of radiation is approximately 10 minutes, or Δ*t* = 0.007 (days), and use the linear model for the radiation $\gamma =\alpha \dot{D}$, where *α* is the radiobiological parameter and $\dot{D}$ is the dose rate. For *α* = 0.08 (1/Gy) (taken from an average of the values obtained in [17]) and the standard dose rate of 5 (Gy per radiation time), we obtain *γ* = 60 (1/day). Using the parameter values *C* = 2.5 (corresponding to the maximum allowed dose rate of 5 (Gy per radiation time)), *F*′ = 25 (mm^2^), and a range of (*n*_0_, *σ*) pairs chosen such that *N*(*t* = *t*_0_) = 10^7^ (cells) is constant, we can solve for *r*_1_ and calculate the corresponding *f*_1_. The location of the optimal radius *r*_1_ is plotted in S1 Fig. As expected, larger values of *σ* require radiation over a wider radius. However, the increase of *r*_1_ with *σ* is sub-linear, and quite well fitted by *r*_1_ ∝ *σ*^1/2^. This is precisely the scaling behavior predicted in Eq (13) for the semi-circular beam shape in *d* = 2. The scaling of the optimal radius with tumour size thus appears to be robust, irrespective of the beam shape. This procedure is done for 5 different (*n*_0_, *σ*) pairs in Table 2 and the profiles can be seen in S3 Fig (Supporting Information). Note that for the *σ* = 1 and *σ* = 2 cases, the optimal *r*_1_ falls below $\sqrt{10}$ (mm). However, due to our constraints of $F^{\prime }=25=f_{1}r_{1}^{2}$ and 0 ≤ *f*(*r*, *t*) ≤ *C* = 2.5, we have a lower bound of $r_{1}\geq \sqrt{10}$ (mm). Also note that the calculated values in Table 2, and in the following data tables, are truncated decimals, but the values of *N* are calculated using more precise solutions. Therefore, inserting the values for *r*_1_ and *f*_1_ found from Table 2 into Eq (20) will not necessarily exactly reproduce the listed values of *N*.

**Table 2:** One fraction & one radial step.

*σ* (mm)	*n*_0_	*r*_1_ (mm)	*f*_1_	*N*(*t* + *dt*)
1	1.59e6	$\sqrt{10}$	2.50	3.54e6
2	3.98e5	$\sqrt{10}$	2.50	5.36e6
3	1.78e5	3.71	1.82	7.16e6
4	1.04e5	4.28	1.36	8.01e6
5	7.36e4	4.79	1.09	8.43e6

Optimal one-step profile for one fraction of radiation with exponential growth and death.*n*_0_ and *σ* are the magnitude and standard deviation of the initial Gaussian tumour cell density, which starts with 10^7^ total cells. *r*_1_ and *f*_1_ are the radius and strength of the step in the cytotoxic radiation profile. *N*(*t* + *dt*) is the final tumour cell number at the end of radiation.

#### D2: Two-step radial profile

Now we consider the more elaborate example of a 2-step cytotoxic profile, but still applied in only one fraction. Introducing two new variables, *r*_2_ and *f*_2_, the radiation profile is:

$$\begin{array}{c} f\left(r,t_{0}\leq t\leq t_{0}+\Delta t\right)=\{\begin{array}{cc} f_{1} & 0\leq r\leq r_{1},\\ f_{2} & r_{1}<r\leq r_{2},\\ 0 & r_{2}<r\leq R. \end{array} \end{array}$$ (26)

where *f*_2_ applies a different dosage to the outer region of the tumour. Adding the second radial arc modifies the constraint on dosage to

$$\begin{array}{c} F^{\prime }=\frac{F_{1}}{dt}=f_{1}r_{1}^{2}+f_{2}\left(r_{2}^{2}-r_{1}^{2}\right). \end{array}$$ (27)

We need to minimize the total cell number, *N*, with respect to the four-parameter set (*r*_1_, *r*_2_, *f*_1_, *f*_2_). Using the Lagrange multiplier method as above results in a system of 5 coupled transcendental equations. Unfortunately, this set of equations has many local extrema, making the result heavily dependant on the initial guess used in the computational solver. This makes identification of the global extreme difficult. To avoid this, we use a Monte Carlo method to identify the optimal shape of the radiation beam. First, a random radiation beam is generated by selecting a point from the parameter space (*r*_1_, *r*_2_, *f*_1_, *f*_2_). The selection of this point is made by randomly assigning values to 3 of the parameters within their acceptable ranges, then calculating the fourth according to Eq (27) such that the dose constraint is satisfied. This generated parameter set defines the candidate radiation beam. The total cell number resulting from each beam is calculated and compared to the previous minimum. This is done many times (∼10^9^ in our simulations) such that *N* converges to a global minimum. The point in the parameter space which generates this minimum is then inserted back into the equations for the optimal profile to check that they are indeed satisfied. Such a point parameterizes the optimal profile (in subsequent optimizations the parameter space will become more complex, however this method will remain the same). The numerical values resulting from this method are summarized in Table 3, and the resulting cell density profiles are shown in S4 Fig (Supporting Information). As a check on the numerical optimization, we do find that the the addition of the second radial arc leads to better treatment outcomes. Once the constraint on maximal value of *f*_1_ = 2.5 is no longer operative (for *σ* > 2 mm), the resulting cell density profiles are close approximations to the flat-top profiles expected from a semi-circular beam. The optimal two step radial profile thus represents a crude approximation to the semi-circular beam.

**Table 3:** One fraction & two radial steps.

*σ* (mm)	*r*_1_ (mm)	*r*_2_ (mm)	*f*_1_	*f*_2_	*N*(*t* + *dt*)
1	3.06	3.27	2.50	1.18	3.54e6
2	2.81	3.47	2.50	1.27	5.30e6
3	2.81	3.98	2.10	1.06	7.10e6
4	3.26	4.61	1.57	0.78	7.98e6
5	3.64	5.15	1.26	0.64	8.41e6

Optimal two-step profile for one fraction of radiation with exponential growth and death. *σ* is the standard deviation of the initial Gaussian tumour cell density, which starts with 10^7^ total cells. *r*_1_, *r*_2_, *f*_1_ and *f*_2_ are the two radii and strengths of the steps in the cytotoxic radiation profile. *N*(*t* + *dt*) is the final tumour cell number at the end of radiation.

#### D3: Two fractions individually constrained

We next consider application of a second fraction of radiation, with dosage separately constrained on each fraction. Describing the second fraction requires introducing four new variables to fully parametrize *f*(*r*, *t*). We modify the previous notation by adding a second index to each of the radii and strengths of *f*(*r*, *t*), with the first index indicating the step-number and the second corresponding to the fraction number. Thus, *r*_1_, *r*_2_, *f*_1_, and *f*_2_ from before become *r*_11_, *r*_21_, *f*_11_, and *f*_21_ respectively, with corresponding *r*_12_, *r*_22_, *f*_12_, and *f*_22_ for the second fraction. The interval between the two fractions will be labelled *τ*, such that the second fraction takes place over the interval [*t*_0_ + Δ*t* + *τ*, *t*_0_ + 2Δ*t* + *τ*]. Note that we assume the lengths of the two fractions to be the same. Defining $t_{0}^{*}\in \left[t_{0},t_{0}+\Delta t\right]$ and $t_{1}^{*}\in \left[t_{0}+\Delta t+\tau ,t_{0}+2\Delta t+\tau \right]$, we can write *f*(*r*, *t*) during the separate fractions as

$$\begin{array}{c} f\left(r,t_{0}^{*}\right)=\{\begin{array}{cc} f_{11} & 0\leq r\leq r_{11}\\ f_{21} & r_{11}<r\leq r_{21}\\ 0 & r_{21}<r\leq R \end{array},\;\text{and}\;f\left(r,t_{1}^{*}\right)=\{\begin{array}{cc} f_{12} & 0\leq r\leq r_{12}\\ f_{22} & r_{12}<r\leq r_{22}\\ 0 & r_{22}<r\leq R \end{array}. \end{array}$$ (28)

In this section, we consider the fractions constrained individually, such that

$$\begin{array}{c} F^{\prime }=f_{11}r_{11}^{2}+f_{21}\left(r_{21}^{2}-r_{11}^{2}\right),\;\text{and}\;F^{\prime }=f_{12}r_{12}^{2}+f_{22}\left(r_{22}^{2}-r_{12}^{2}\right). \end{array}$$ (29)

Since our constraint on $f(r,t_{0}^{*})$ is the same as before, the optimal $f(r,t_{0}^{*})$ does not change from that in Table 2. We can use the same optimization procedure for the second fraction, but with a modified starting cell density profile. In order to deal with profiles with simple analytic expression, we further assume that the time interval *τ* between the fractions is small enough to neglect spatial migrations described by the diffusion term (mathematically, this can be expressed as the condition *D*_*n*_ *τ* ≪ *σ*^2^, which can be derived from a simple scale analysis). If so, the density profile simply grows exponentially, by a factor *e*^*ρτ*^ without changing its spatial form, and immediately before the second fraction is given by

$$\begin{array}{c} n\left(r,t_{0}+dt+\tau \right)=\{\begin{array}{cc} n_{0}e^{\rho \tau }e^{-\frac{r^{2}}{2\sigma^{2}}}e^{-\gamma f_{11}\Delta t}\; & 0\leq r\leq r_{11}\\ n_{0}e^{\rho \tau }e^{-\frac{r^{2}}{2\sigma^{2}}}e^{-\gamma f_{21}\Delta t} & r_{11}<r\leq r_{21}\\ n_{0}e^{\rho \tau }e^{-\frac{r^{2}}{2\sigma^{2}}} & r_{21}<r\leq R \end{array}. \end{array}$$

The density profile immediately after application of the second fraction is then given by:

$$\begin{array}{c} n\left(r,t_{0}+2dt+\tau \right)=\{\begin{array}{cc} n\left(r,t_{0}+dt+\tau \right)e^{-\gamma f_{12}\Delta t} & 0\leq r\leq r_{12}\\ n\left(r,t_{0}+dt+\tau \right)e^{-\gamma f_{22}\Delta t} & r_{12}<r\leq r_{22}\\ n(r,t_{0}+dt+\tau ) & r_{22}<r\leq R \end{array}. \end{array}$$

For each set of radii, *r*_11_, *r*_21_, *r*_12_, and *r*_22_, the cell density is a piecewise continuous function. The total number *N* can then be obtained as before by integration, as an explicit analytic expression. For the same initial combinations of (*n*_0_, *σ*) as above, we search for the radii (*r*_21_, *r*_22_) that optimize the second fraction using our Monte Carlo method (the optimal radii (*r*_11_, *r*_12_) are naturally the same as obtained previously in Table 3). The results of this minimization are summarized in Table 4.

**Table 4:** Two two-step fractions, separately constrained.

*σ*(*mm*)	*r*_11_(*mm*)	*r*_21_(*mm*)	*f*_11_	*f*_21_	*r*_12_(*mm*)	*r*_22_(*mm*)	*f*_12_	*f*_22_	*N*(*T*)
1	3.06	3.27	2.50	1.18	2.93	3.36	2.50	1.30	1.29e6
2	2.81	3.47	2.50	1.27	1.97	4.04	2.42	1.26	3.41e6
3	2.81	3.98	2.10	1.06	4.61	5.03	1.07	0.57	5.59e6
4	3.26	4.61	1.57	0.78	5.36	5.84	0.80	0.39	6.80e6
5	3.64	5.15	1.26	0.64	5.98	6.54	0.64	0.31	7.52e6

Optimal two-step profile for two separately constrained fractions of radiation with exponential growth and death. *σ* is the standard deviation of the initial Gaussian tumour cell density, which starts with 10^7^ total cells. *r*_11_, *r*_21_, *r*_12_, *r*_22_, *f*_11_, *f*_21_, *f*_12_ and *f*_22_ are the radii and strengths of the steps in the cytotoxic radiation profiles. *N*(*t* + *dt*) is the final tumour cell number at the end of radiation.

Note that if diffusion is ignored, the final cell density after a second fraction of radiation is given by $n\left(\vec{x}\right)=n_{0}\left(\vec{x}\right)\;\text{exp}\;\{\rho \tau -\gamma [f_{1}\left(\vec{x}\right)+f_{2}\left(\vec{x}\right)]\Delta t\}$. From this expression it follows that in this limit, the order in which the two fractions are applied is not important. Indeed the same conclusion applies to any number of fractions, each separately optimized. Thus it is not necessary to use the XRT profile that is optimal at the time of its application, as long as there are planned future fractions that boost the overall amount of radiation at each point to the optimal value. This freedom provides an additional tool for therapeutic planning.

#### D4: Two fractions with overall constraint

As a final example within this class, with non-diffusive exponential growth between the two fractions, we consider the case when the overall dose is constrained, i.e. as

$$\begin{array}{c} 2F^{\prime }=f_{11}r_{11}^{2}+f_{21}\left(r_{21}^{2}-r_{11}^{2}\right)+f_{12}r_{12}^{2}+f_{22}\left(r_{22}^{2}-r_{12}^{2}\right). \end{array}$$

The optimization procedure can be carried out as before through a Monte Carlo search. However, each step of the search now involves an exploration in the space of 8 variables (as opposed to separate searches in a space of 4 variables), subject to one constraint. Note also that (except for the replacement of 2*F*′ for *F*′) this is exactly the search that would be performed for a single fraction with a 4-step profile. The optimal values of these parameters are given in Table 5.

**Table 5:** Two two-step fractions, mutually constrained.

*σ* (mm)	*r*_11_ (mm)	*r*_21_ (mm)	*f*_11_	*f*_21_	*r*_12_ (mm)	*r*_22_ (mm)	*f*_12_	*f*_22_	*N*(*T*)
1	3.06	3.27	2.50	1.18	2.93	3.36	2.50	1.30	1.29e6
2	2.81	3.47	2.50	1.27	1.97	4.04	2.42	1.26	3.41e6
3	3.55	4.92	1.63	0.82	2.56	4.31	1.61	0.78	5.59e6
4	4.20	5.74	1.21	0.61	2.97	4.92	1.22	0.55	6.79e6
5	4.63	6.34	1.02	0.50	3.28	5.52	0.96	0.43	7.42e6

Optimal two-step profile for two mutually constrained fractions of radiation with exponential growth and death. *σ* is the standard deviation of the initial Gaussian tumour cell density, which starts with 10^7^ total cells. *r*_11_, *r*_21_, *r*_12_, *r*_22_, *f*_11_, *f*_21_, *f*_12_ and *f*_22_ are the radii and strengths of the steps in the cytotoxic radiation profiles. *N*(*t* + *dt*) is the final tumour cell number at the end of radiation.

Observe that for each *σ*, the optimal *N* with the single overall constraint is either better, or the same as, in the individually constrained case. From the perspective of optimization, this is not unexpected as the latter also explores the subset of the space available to the former. In view of this, the surprising result may appear to be that for *σ* = 1 and 2 (see Table 6) the minimum occurs in the separately constrained space. However, the structure of the general optimization problem is such that for *D*_*n*_ = 0, the same result should hold with one or more constraints (supporting infomation). We are thus unable to conclude if the unequal partition of flux between the two fractions (in cases of *σ* = 3, 4, 5) is correct or a computational artifact (we indeed find many solutions close to the optimum, so finding the true optimum requires considerable computation and precision).

**Table 6:** Variation of flux over two fractions.

*σ* (mm)	1st Fraction	2nd Fraction
1	25.00	25.00
2	25.00	25.00
3	30.08	19.92
4	30.77	19.23
5	31.27	18.73

Distribution of radiation flux over two radiation fractions based on standard deviation of initial tumour cell density. The ‘1st Fraction’ number represents the amount of dose (out of 50) that is allocated to the 1st radiation fraction and the ‘2nd fraction’ the amount allocated to the 2nd radiation fraction.

#### D5: Tumour densities from logistic growth

The Gaussian density profiles employed to model the initial cell densities above are appropriate only for small undeveloped tumours. For contrast, in this section we consider density profiles resulting from the logistic growth (*a* = 1 in Eq (1)). Such profiles are obtained by evolving the partial differential equation

$$\begin{array}{c} \frac{\partial n}{\partial t}=D_{n}\nabla^{2}n+\rho n\left(1-\frac{n}{n_{max}}\right), \end{array}$$ (30)

starting with a localized initial condition. While there is no exact analytic form for the resulting solution, as an analytical approximant, we use the form

$$\begin{array}{c} n\left(r,t_{0}\right)=\frac{a_{1}}{1+b_{1}\;e^{c_{1}\;r^{2}}}, \end{array}$$ (31)

where *a*_1_, *b*_1_, and *c*_1_ are fit parameters. Logistic growth causes the tumour to form a flat-top profile as the density in the centre approaches *n*_*max*_. Eq (31), which is known as a Fermi Function, also describes a flat-top form, which is why we use it for fitting. A numerical implementation of Eq (1) was used to generate the fit parameters. For this procedure and more information, see supporting information. The values of the fitted parameters are given in Table 7.

**Table 7:** Fitted parameters as in Eq (31).

*σ*	*a*_1_	*b*_1_	*c*_1_
1	2.0697e6	0.4237	0.04007
3	1.0978e6	-0.3069	-0.004395
5	1553387	-0.02081	-0.01505

Fitting parameters for initial density generated by simulation with logistic growth. Parameters correspond to those in Eq (31).

We consider the same radial step-functions as previous, and the optimization procedures are carried out in the same manner as before. The results for application of a single 2-step fraction are reported in Table 8. If this treatment is followed by a second, separately constrained 2-step fraction, the resulting parameters for the second application are given in Table 9.

**Table 8:** One fraction & two radial steps for logistic growth.

*σ*	*r*_1_ (mm)	*r*_2_ (mm)	*f*_1_	*f*_2_
1	3.89	5.23	1.16	0.61
3	6.73	10.00	0.34	0.17
5	5.80	10.00	0.26	0.24

Optimal one-step profile for one fraction of radiation with logistic growth and exponential death. *σ* is the standard deviation of the initial Gaussian tumour cell density, which starts with 10^7^ total cells. *r*_1_, *r*_2_, *f*_1_, and *f*_2_ are the radii and strengths of the steps in the cytotoxic radiation profiles. *N*(*t* + *dt*) is the final tumour cell number at the end of radiation.

**Table 9:** Two two-step fractions, each separately constrained for logistic growth.

*σ*	*r*_11_	*r*_21_	*f*_11_	*f*_21_	*r*_12_	*r*_22_	*f*_12_	*f*_22_
1	3.89	5.23	1.16	0.61	5.92	6.37	0.66	0.33
3	6.73	10.00	0.34	0.17	3.98	10.00	0.31	0.24
5	5.81	10.00	0.26	0.24	8.47	10.00	0.25	0.25

Optimal one-step profile for one fraction of radiation with logistic growth and exponential death. *σ* is the standard deviation of the initial Gaussian tumour cell density, which starts with 10^7^ total cells. *r*_11_, *r*_21_, *r*_12_, *r*_22_, *f*_11_, *f*_21_, *f*_12_ and *f*_22_ are the radii and strengths of the steps in the cytotoxic radiation profiles. *N*(*t* + *dt*) is the final tumour cell number at the end of radiation.

#### D6: Tumour densities from logistic growth and logistic death

The previously examined cases of exponential cytotoxic action do not incorporate the phenomenon of radiation having a larger effect on faster-proliferating cells than on slower-proliferating cells due to the cell’s position in the cell cycle and factors such as oxygen concentration [22]. If we instead consider logistic action (*b* = 1), then some aspects of such tendency are reproduced. This qualitative behavior is important to incorporate because the vast majority of tumours exhibit some form of treatment resistance. This resistance is conferred to a tumour through heterogeneities of various traits across its volume, such as cell-type, phenotypic expression, and stem-ness. Of particular interest to radiation therapy, tumour hypoxia is a prominent feature that leads to increased radioresistance. As we will see, inclusion of logistic cytotoxic action reproduces this behavior and dramatically changes the results. We note that this is a purely phenomenological effect and not meant to describe the underlying biology at play. We make the same simplifications as before leading to the piecewise equivalent to Eq (5),

$$\begin{array}{c} n\left(r,t_{0}+\Delta t\right)=\{\begin{array}{cc} \frac{a_{1}n_{max}e^{-\gamma f_{1}\Delta t}}{a_{1}\left(e^{-\gamma f_{1}\Delta t}-1\right)+n_{max}\left(1+b_{1}e^{c_{1}r^{2}}\right)} & 0\leq r\leq r_{1}\\ \frac{a_{1}n_{max}e^{-\gamma f_{2}\Delta t}}{a_{1}\left(e^{-\gamma f_{2}\Delta t}-1\right)+n_{max}\left(1+b_{1}e^{c_{1}r^{2}}\right)} & r_{1}<r\leq r_{2}\\ \frac{a_{1}}{1+b_{1}e^{c_{1}r^{2}}} & r_{2}<r\leq R. \end{array} \end{array}$$

The results of the Monte Carlo optimization are summarized in Table 10.

**Table 10:** One two-step fraction, logistic death.

*σ*	*r*_1_	*r*_2_	*f*_1_	*f*_2_
1	4.3933	5.7914	0.0000	1.7557
3	9.4731	9.9886	0.0005	2.4875
5	9.4774	9.9906	0.0009	2.4940

Optimal two-step profile for one fraction of radiation with logistic growth and death. *σ* is the standard deviation of the initial Gaussian tumour cell density, which starts with 10^7^ total cells. *r*_1_, *r*_2_, *f*_1_, and *f*_2_ are the radii and strengths of the steps in the cytotoxic radiation profiles. *N*(*t* + *dt*) is the final tumour cell number at the end of radiation.

Mathematically, the results here differ dramatically from the exponential case in which the optimal profile focused the radiative strength in the center of the tumour where the cell-density was larger. With the incorporation of logistic cell death, the effectiveness of radiation in the center of the tumour diminishes. The optimal profile must now balance this with still attacking areas with high cell densities. We see that in the case with the smallest initial spread of cells, the optimal cytotoxic profile achieves this by focusing strength in a ring through the middle of the tumour. For the other two cases, the density was far more spread out initially, leading to an optimal profile which focused on cells at the outside of the tumour. This was because the effect of the logistic death was stronger than the decreased cell density.

In the case of exponential death, we were able to continue our discussion and optimize the second fraction as we did the first. In the logistic death case, the computations in the optimization method become significantly more challenging. For that reason, we do not pursue the second fraction of radiation here.

Table 11 shows a summary of the discrete optimization cases undertaken in this work. It outlines the method, constraints, and details of each of the six cases.

**Table 11:** Summary of discrete optimization cases.

Subsection	Dose Profile	# Fractions	Dose Constraint	Growth	Death	Optimization Method
D1	One-Step	1	*F*_1_	Exponential	Exponential	Lagrange Multiplier
D2	Two-Step	1	*F*_1_	Exponential	Exponential	Monte Carlo
D3	Two-Step	2	*F*_1_ and *F*_2_	Exponential	Exponential	Monte Carlo
D4	Two-Step	2	*F*_1_ + *F*_2_	Exponential	Exponential	Monte Carlo
D5	Two-Step	1	*F*_1_	Logistic	Exponential	Monte Carlo
D6	Two-Step	1	*F*_1_	Logistic	Logistic	Monte Carlo

A summary of the discrete optimization cases undertaken herein.

## Conclusion and discussion

In this paper we pose the question of how to spatially shape a sequence of XRT treatments to best eliminate tumour cells. To answer this question, we need to know **(i)** how the tumour grows in time; **(ii)** how it responds to treatment; and **(iii)** what constraints apply to radiation dosage. Answers to all questions need to be expressed in mathematical terms, which necessitates simplifications and approximations. We have relied on assumptions and mathematical models commonly used in the literature, and hope our general results are insensitive to choice of model.

The most important result follows from the assumption that the effect of XRT is to destroy a fraction of existing tumour cells, proportional to its strength. This assumption is embodied by the term $-\gamma f\left(\vec{x},t\right)n\left(1-bn/n_{max}\right)$ in Eq (2). It immediately leads to the conclusion that the optimal beam profile should depend non-uniformly on the tumour density at the time of its application. Shaping the beam to such a precise form is likely impossible, and may further violate constraints on the strength of the beam. As such, optimal beam profiles can be sought within certain constraints on the shape of the beam. In particular, we consider XRT profiles obtained by superposing several radially symmetric beams. Clinically, the normal procedure is to irradiate the entire radius of the visible tumour with a uniform beam. As can be seen from the various tables above, a uniform *f*(*r*, *t*) will not lead to optimal cell kill. Using a spatially optimized *f*(*r*, *t*) can reduce the total number of cells within a tumour significantly from what is currently done clinically.

The metric of TCP is not the only quantity that can be used to measure radiation efficacy. Others such as normal tissue complication probability (NTCP), conformity index, homogeneity index, and survival time have been used by other researchers [23–26]. We focused our analysis here on TCP as it is intuitively simple and mathematically straightforward. We stress that this is an exploratory study meant to discern qualitative behaviour, and note that a more complete study should include analysis and comparison of other methods of radiation treatment planning. More work in this direction would be interesting and similar mathematical tools could theoretically be used.

For more than one fraction of XRT, it is necessary to account for the growth of the tumour in between the two treatments. A simple commonly used model is logistic growth, depending on three parameters *ρ*, *D*_*n*_ and *n*_max_. In the various studies above, we have mostly neglected the change in the shape of the tumour between two fractions. However, this needs to be included in a more comprehensive study. Given all our results, we can propose a procedure for spatial optimization of radiation as follows:

- Image tumour twice. The change in shape of the tumour can then be used to model its growth mode; e.g. in order to deduce the parameters *ρ*, *D*_*n*_, and *n*_max_. The most important ingredient for shaping the optimal beam is the cell density $n(\vec{x})$.
- Determine dose prescription and treatment schedule for the tumour. This provides constraints on individual and total allowed dosage, {*F*_*n*_} and the time intervals between fractions, {*τ*_*n*_}.
- Determine the physical limitations of the radiation apparatus; e.g. how many radial steps is the apparatus capable of accurately producing and superposing.
- Optimize the first radiation fraction using the above optimization procedure.
- Use the growth model deduced from the initial two images to model the growth of the tumour cells between fractions. The deduced cell density profile prior to each fraction can then be used as input to optimize the shape of the beam in that fraction. The assumptions on evolution of the profile after a fraction and in between fractions are likely to introduce errors. Ideally, more imaging can be done to obtain more precise cell density profiles at intermediate times.

This exploratory work examines spatial variations in the shape of radiation beams, and proposes improvements to radiation therapy by shaping beams according to the pre-treatment cell-density. The advantage of this method is the opportunity to reduce the total tumour cell number below that obtained in uniform radiation. In addition to the initial tumour density profile, the method relies only on few tumour-specific parameters in order to carry out the optimization. In its current implementation, the method does not take into account other factors in radiation planning such as nearby organs at risk, scheduling, or other scoring indices used to compare dose distributions. Such factors could potentially be included in a more elaborate model, a direction that we hope to pursue in future. A major limitation of the model is the exclusion of heterogeneity across the tumour volume as tumours usually vary in cell type, stemness, phenotypic expression, and perhaps most importantly, hypoxia. Although we added a logistic cytotoxic action term to account for different radiation efficacy across the area, this is relatively simplistic and could be improved with a more thorough specification of tumour heterogeneity. Another direction that was not examined here is the interaction of this spatially-varying radiation treatment with other therapies such as chemotherapy. We hope that this exploratory work sheds light on the spatial variation of radiation beams and inspires further work in the area.

## Supporting information

S1 File Supporting information and figures. (PDF)

S1 Fig Optimal beam radius *r*_1_ vs. tumour size *σ* from Eq (25). (EPS)

S2 Fig Total cells after first fraction vs. radius of step in cytotoxic profile. The solid lines correspond to different values of *σ* with red for *σ* = 1, blue for *σ* = 2, green for *σ* = 3, teal for *σ* = 4, and purple for *σ* = 5. The dots correspond to individual runs of the pseudo-spectral method.
(EPS)

S3 Fig Cell densities from one one-step fraction with of exponential growth and death. Cell densities resulting from the optimal radiation profiles from one 1-step fraction of exponential growth and death for the initial density deviations of (from top left to bottom right) *σ* = 1, *σ* = 2, *σ* = 3, *σ* = 4, and *σ* = 5. The red dots are those below the detectable threshold in imaging, of 5 cells/mm^2^, and the blue dots those above. Note the different y-axis scales to highlight the resulting shape.
(EPS)

S4 Fig Cell densities from two one-step fractions of exponential growth and death. Cell densities resulting from the optimal radiation profiles from one 2-step fraction of exponential growth and death for the initial density deviations of (from top left to bottom right) *σ* = 1, *σ* = 2, *σ* = 3, *σ* = 4, and *σ* = 5. The red dots are those below the detectable threshold in imaging, of 5 cells/mm^2^, and the blue dots those above. Note the different y-axis scales to highlight the resulting shape.
(EPS)

S5 Fig Cell densities from one two-step fraction of exponential growth and death. Cell densities resulting from the optimal radiation profiles from two 2-step fraction of exponential growth and death for the initial density deviations of (from top left to bottom right) *σ* = 1, *σ* = 2, *σ* = 3, *σ* = 4, and *σ* = 5. The red dots are those below the detectable threshold in imaging, of 5 cells/mm^2^, and the blue dots those above. Note the different y-axis scales to highlight the resulting shape.
(EPS)

S6 Fig Cell densities from two two-step fractions of exponential growth and death. Cell densities resulting from the optimal radiation profiles from two 2-step fraction of exponential growth and death for the initial density deviations of (from top left to bottom right) *σ* = 1, *σ* = 2, *σ* = 3, *σ* = 4, and *σ* = 5. The red dots are those below the detectable threshold in imaging, of 5 cells/mm^2^, and the blue dots those above. Note the different y-axis scales to highlight the resulting shape.
(EPS)

S7 Fig Cell densities from one two-step fractions of logistic growth and exponential death. Cell densities resulting from the optimal radiation profiles from one 2-step fraction of logistic growth and exponential death for the initial density deviations of (from left to right) *σ* = 1, *σ* = 3, and *σ* = 5. The red dots are those below the detectable threshold in imaging, of 5 cells/mm^2^, and the blue dots those above. Note the different y-axis scales to highlight the resulting shape.
(EPS)

S8 Fig Cell densities from two two-step fractions of logistic growth and exponential death. Cell densities resulting from the optimal radiation profiles from two 2-step fractions of logistic growth and exponential death of (from left to right) *σ* = 1, *σ* = 3, and *σ* = 5. The red dots are those below the detectable threshold in imaging, of 5 cells/mm^2^, and the blue dots those above. Note the different y-axis scales to highlight the resulting shape.
(EPS)

S9 Fig Cell densities from one two-step fractions of logistic growth and death. Cell densities resulting from the optimal radiation profiles from one 2-step fraction of logistic growth and death of (from left to right) *σ* = 1, *σ* = 3, and *σ* = 5. The red dots are those below the detectable threshold in imaging, of 5 cells/mm^2^, and the blue dots those above. Note the different y-axis scales to highlight the resulting shape.
(EPS)
