# Supplementary material for: Spatial optimization for radiation therapy of brain tumours
Source: PLoS One. 2019 Jun 28;14(6):e0217354. doi: 10.1371/journal.pone.0217354 (PMC6599149; doi:10.1371/journal.pone.0217354)
Supplement: S1 File — (PDF) [file pone.0217354.s001.pdf]

Supporting information

Proof of uniform resulting cell density for arbitrary death mechanism

We have seen in the previous sections that the resulting cell density profile after one fraction of radiation is uniform for both exponential and logistic death. However, one might wonder whether this result holds for any arbitrary death mechanism. In fact, this is the case, and for any arbitrary radiation death mechanism, $g(n(r,t))$, the cytotoxic profile which minimizes the total number of surviving cells results in a uniform cell density.

To show this, we first modify Eq. (5) to include the arbitrary death mechanism

$$\frac{\partial n}{\partial t} = -\gamma f(r) g(n(r,t)),$$

where $g(n(r,t))$ is the death mechanism (ie. exponential, logistic, or other). Note that we are still limited here to the death rate being proportional to $f(r,t)$; we suspect that this result is not true for arbitrary functions of $f(r,t)$. Integrating this equation with respect to t over the fraction (total time of Δt) yields:

$$\int_{n_0(r)}^{n_1(r)} \frac{dn}{g(n(r,t))} = -\gamma \Delta t f(r).$$

We now differentiate this equation with respect to f, using the Fundamental Theorem of Calculus, and the fact that $\frac{\partial n_0}{\partial f} = 0$ (since the initial cell density is of course independent of our radiation profile). This leaves us simply with

$$\frac{\partial n_1}{\partial f} \frac{1}{g(n_1(r))} = -\gamma \Delta t. \tag{1}$$

Then, from our optimization constraint (equivalent to Eq. (8))

$$\tilde{N_1} = \int d^d\vec{x}\; n_1(r) + \lambda \Big(\int d^d\vec{x}\; f(\vec{x}, t_0) - F \Big),$$

we can use the Euler-Lagrange Equation to arrive at the constraint on f of

$$\frac{\partial n_1}{\partial f} + \lambda = 0.$$

Substituting this into Eq. (1) above and rearranging gives:

$$g(n_1) = \frac{\lambda}{\gamma \Delta t}.$$

Since λ, γ, and Δt are all constants, and $g(n_1(r))$ only explicitly depends on $n_1(r)$, this equation can be rearranged to solve for $n_1(r)$ as a constant for any arbitrary and invertible function $g(n(r,t))$.

Thus, the continuous profile which minimizes the total number of surviving cells will always yield a uniform surviving cell density.

Optimal profile with two fractions of exponential death

The optimal XRT profile in the first fraction naturally does not depend on the tumour growth mechanism. However, it is reasonable to expect that the additional growth of

the tumour prior to application of the second fraction will lead to joint optimal profiles that do. Since it it difficult to treat the full non-linear (logistic) growth, we make the further assumption that the growth process in the interval between the two fractions is exponential (this will be the case if the initial fraction reduces the density to well below n_{max}, and the interval between the two fractions is short enough for $n(\vec{x}, t)$ to remain smaller than this saturation value). Formal integration of the linear partial differential equation now leads to the following cell density profile after a time $t_1 = t_0 + \tau$ since application of the first fraction

$$n(\vec{x}, t_1) = \exp\left[\tau\left(\rho + D_n \nabla^2\right)\right] * \left[e^{-f(\vec{x}, t_0)} n(\vec{x}, t_0)\right], \tag{2}$$

where $*$ indicates a convolution of the 2 functions and τ is the time between fractions.

If the resulting cell density from the first fraction was uniform, then Eq. (2) will simply leave us with another uniform distribution, in which case, the optimal radiation fraction is, of course, uniform. However, if the optimal first fraction was cut-off as in the semi-circular example above, then we will have to take more care in the application of our second fraction. Leaving the cell density general, following the application of a second fraction, the cell density is

$$n(\vec{x}, t_1 + \Delta t) = e^{-f_2(\vec{x})} \exp\left[\tau\left(\rho + D_n \nabla^2\right)\right] * \left[e^{-f_1(\vec{x})} n(\vec{x}, t_0)\right], \tag{3}$$

where we have used $f_1(\vec{x}) \equiv f(\vec{x}, t_0)$ and $f_2(\vec{x}) \equiv f(\vec{x}, t_1)$. Using properties of the diffusion operator, the total number of tumour cells after the second fraction can be written as

$$N_2 = e^{\rho\tau} \int \frac{d^d\vec{x} d^d\vec{x'}}{(2\pi D\tau)^{d/2}} \left(e^{-f_2(\vec{x})}\right) e^{-\frac{(\vec{x'} - \vec{x})^2}{2D\tau}} \left(e^{-f_1(\vec{x'})} n(\vec{x'}, t_0)\right). \tag{4}$$

Minimizing the above expression, we arrive at the conditions

$$\left[e^{-f_2(\vec{x})}\right] e^{\tau D_n \nabla^2} \left[e^{-f_1(\vec{x})} n(\vec{x}, t_0)\right] = \lambda_2 e^{-\rho\tau}, \tag{5}$$

$$\left[e^{-f_1(\vec{x})} n(\vec{x}, t_0)\right] e^{\tau D_n \nabla^2} \left[e^{-f_2(\vec{x})}\right] = \lambda_1 e^{-\rho\tau}. \tag{6}$$

Here, λ_1 and λ_2 are distinct Lagrange multipliers to impose separate constraints on the total flux in each fraction. If the constraint acts only on the sum of the two fractions, then $\lambda_1 = \lambda_2 = \lambda$. Actually, the equations are symmetric with respect to exchange of the functions $\Phi_1(\vec{x}) \equiv e^{-f_1(\vec{x})} n(\vec{x}, t_0)$ and $\Phi_2(\vec{x}) \equiv e^{-f_2(\vec{x})} n(\vec{x}, t_0)$, suggesting solutions of the form $\Phi_1(\vec{x}) = \Phi_2(\vec{x})$ implying $\lambda_1 = \lambda_2$, i.e. constraining radiation in each fraction, or in the sum, would correspond to the same optimal solution. In the absence of other constraints, the above equations are solved by $\nabla^2\Phi_1 = \nabla^2\Phi_2 = 0$, i.e. position-independent Φ_1 and Φ_2. This again leads to $f_1 = \ln(n_0/\lambda)$ (as in Eq. (9)), albeit for a different reason) followed by a uniform f_2. This general solution is not particularly useful and may not be viable given various constraints.

Additional figures and simulations

In addition to the optimization procedure outlined in the main text of the paper, Eq. (2) was also implemented numerically using a pseudo-spectral method. Using this implementation, the results from the optimization procedure can be checked. As an illustrative example of this, take the case of the 1-step cytotoxic profile with logistic growth and death. Using the psuedo-spectral implementation, Eq. (2) (with $a = b = 0$) was simulated for many different forms of $f(r, t)$ and the total cell number N calculated

immediately after the fraction. The $f(r,t)$ that produced the minimum cell number from this method was obtained and compared to the optimal $f(r,t)$ from the procedure in the main text. S2 Fig shows some justification of the agreement of the 2 methods on the optimal profile.

From this numerical method, the cell-density profiles were also obtained so that the results can be better visualized. These figures are shown in S3 and S9 Figs.

For simulations we employ values of $\rho = 0.35$ (1/day) and $D_n = 0.32$ (mm^2/day) in Eq. (1), in accord with common works and previous measurements [20], although the results are not sensitive to this choice.

For cases with logistic growth, we cannot use a Gaussian as our initial profile. Logistic growth causes the tumour to form a flat-top profile as the density in the centre approaches n_{max}. Since Eq. (1) has no analytical solution for logistic growth, we instead use a numerical technique to find the initial profile. Starting with initial profiles in the form of normal distributions, with widths $\sigma = 1$, 3, and 5, the density profile is simulated with logistic growth for a time of 12.5 days. The resulting profile is then fitted to Eq. (35). The resulting fitted parameters are listed in Table 7.
